# Lactobacilli extracellular vesicles: potential postbiotics to support the vaginal microbiota homeostasis

**DOI:** 10.1186/s12934-022-01963-6

**Published:** 2022-11-15

**Authors:** Vanessa Croatti, Carola Parolin, Barbara Giordani, Claudio Foschi, Stefano Fedi, Beatrice Vitali

**Affiliations:** 1 Department of Pharmacy and Biotechnology, University of Bologna, Bologna, Italy; 2 Section of Microbiology, Department of Experimental, Diagnostic and Specialty Medicine, University of Bologna, Bologna, Italy; 3 Microbiology Unit, IRCCS Azienda Ospedaliero-Universitaria Di Bologna, Bologna, Italy

**Keywords:** Extracellular vesicles, *Lactobacillus*, Adhesion, Postbiotics, Vaginal homeostasis

## Abstract

**Background:**

*Lactobacillus* species dominate the vaginal microflora performing a first-line defense against vaginal infections. Extracellular vesicles (EVs) released by lactobacilli are considered mediators of their beneficial effects affecting cellular communication, homeostasis, microbial balance, and host immune system pathways. Up to now, very little is known about the role played by *Lactobacillus* EVs in the vaginal microenvironment, and mechanisms of action remain poorly understood.

**Results:**

Here, we hypothesized that EVs can mediate lactobacilli beneficial effects to the host by modulating the vaginal microbiota colonization. We recovered and characterized EVs produced by two vaginal strains, namely *Lactobacillus crispatus* BC5 and *Lactobacillus gasseri* BC12. EVs were isolated by ultracentrifugation and physically characterized by Nanoparticle Tracking Analysis (NTA) and Dynamic Light Scattering (DLS). EVs protein and nucleic acids (DNA and RNA) content was also evaluated. We explored the role of EVs on bacterial adhesion and colonization, using a cervical cell line (HeLa) as an in vitro model. Specifically, we evaluated the effect of EVs on the adhesion of both vaginal beneficial lactobacilli and opportunistic pathogens (i.e., *Escherichia coli, Staphylococcus aureus, Streptococcus agalactiae,* and *Enterococcus faecalis*). We demonstrated that EVs from *L. crispatus* BC5 and *L. gasseri* BC12 significantly enhanced the cellular adhesion of all tested lactobacilli, reaching the maximum stimulation effect on strains belonging to *L. crispatus* species (335% and 269% of average adhesion, respectively). At the same time, EVs reduced the adhesion of all tested pathogens, being EVs from *L. gasseri* BC12 the most efficient.

**Conclusions:**

Our observations suggest for the first time that EVs released by symbiotic *Lactobacillus* strains favor healthy vaginal homeostasis by supporting the colonization of beneficial species and preventing pathogens attachment. This study reinforces the concept of EVs as valid postbiotics and opens the perspective of developing postbiotics from vaginal strains to maintain microbiota homeostasis and promote women’s health.

## Background

The vaginal microbiota of a healthy woman is mainly composed of *Lactobacillus* species which significantly affect the homeostasis of the vaginal ecosystem [1, 2]. *Lactobacillus* colonization of the vaginal mucosa reduces urogenital infections preventing pathogens’ adhesion, attachment, and consequent invasion to host tissues [3, 4]. Moreover, lactobacilli produce a wide range of antimicrobial metabolites such as lactic acid, hydrogen peroxide (H_2_O_2_), lectins, bacteriocins, and biosurfactants, whose activity was demonstrated towards a broad spectrum of pathogens [5–11].

Despite their thick cell wall, *Lactobacillus* spp. can produce extracellular vesicles (EVs), spherical lipid bilayer membrane-derived structures widespread throughout all domains of life [12]. During vesiculogenesis, EVs load different molecules, such as lipids, proteins, nucleic acids, and other compounds from various cell compartments that are exported and, subsequently, available in the environment [13]. Depending on the cargo and the surface composition, EVs are involved in various biological pathways like cell viability, nutrient uptake, antibiotic resistance, nucleic acids transfer, biofilm formation, intraspecies communication (quorum-sensing), and communication with the host (crosstalk) [14].

Regarding *Lactobacillus* spp., EVs play a role in mediating their beneficial effect to the host, affecting pathogen infectivity and/or modulating the host immune system. Indeed, it has been reported that EVs from lactobacilli can reduce pathogen infection by the exposure of antimicrobial molecules and/or by mediating the competitive exclusion between pathogenic and mutualistic bacteria [15–18]. In addition, *Lactobacillus* EVs impaired enterococci and *Staphylococcus aureus* infection by modulating host immune system pathways [18, 19]. As more and more studies highlight the association between EVs and the probiotic bacteria health benefits, the concept of EVs as new postbiotics is coming to consolidation [20].

Besides this evidence, only one study reported the protective role of EVs from *Lactobacillus* in the vaginal niche [8], pointing out the need for further investigations in this field.

In the present paper, we investigated the potential of *Lactobacillus* EVs in modulating the vaginal microbiota composition in favor of the host state of health. EVs were recovered from two *Lactobacillus* strains isolated from the healthy vagina, namely *Lactobacillus crispatus* BC5 and *Lactobacillus gasseri* BC12 [10]. First, we characterized EVs physical and chemical properties in terms of yield, size, and total protein and nucleic acids (DNA and RNA) content. We sought for the ability of EVs to modulate the adhesion of beneficial resident lactobacilli, including the producing strains themselves and other strains belonging to the species *L. crispatus* (BC1, BC3, and BC4) and *L. gasseri* (BC9, BC10, and BC11). Moreover, EVs effects were tested toward the adhesion of four vaginal opportunistic pathogens: *Escherichia coli*, *Staphylococcus aureus*, *Streptococcus agalactiae*, and *Enterococcus faecalis*.

## Results

### Characterization of EVs released by vaginal lactobacilli

EVs were isolated from *L. crispatus* BC5 and *L. gasseri* BC12 strains at the stationary growth phase in MRS medium. EVs concentrations and dimensions were analyzed by NTA technology, Z-potential was measured by DLS technique. EVs were also recovered from sterile MRS medium and physically characterized. Results are reported in Table 1.

**Table 1:** Physical characterization of EVs isolated from *L. crispatus* BC5, *L. gasseri* BC12 and MRS medium

	Concentration (particles/mL)	Size (nm)	Ζ-potential (mV)
*L. crispatus* BC5-EVs	34.70 ± 2.31 × 10^9^	89.3 ± 49.2	− 20.3 ± 1.8
*L. gasseri* BC12-EVs	30.60 ± 1.05 × 10^9^	129.1 ± 51.9	− 10.4 ± 0.7
MRS-EVs	14.40 ± 0.71 × 10^9^	150.9 ± 73.8	− 26.1 ± 3.4

Data are reported as mean ± standard deviation (SD) (n = 2)

As reported in Table 1, *L. crispatus* BC5 and *L. gasseri* BC12 produced EVs in similar amounts, EVs average size slightly varied between the two strains (mean diameter of 89.3 nm vs 129.1 nm). EVs Z-potential resulted in negative values, *L. crispatus* BC5-EVs Z-potential was significantly lower (− 20.3 ± 1.8 mV) than that of *L. gasseri* BC12-EVs (− 10.4 ± 0.7 mV) (*p* < 0.05). Particles were also found in MRS medium not conditioned by microorganisms, in a concentration about 2.5-fold lower than EVs derived from bacteria. EVs from MRS medium displayed larger dimension and significantly lower Z-potential than those reported for *L. crispatus* BC5 and *L. gasseri* BC12 (*p* < 0.05).

EVs samples were also investigated in terms of protein and nucleic acid content and results are reported in Table 2.

**Table 2:** Chemical characterization of EVs isolated from *L. crispatus* BC5, *L. gasseri* BC12 and MRS medium

	Protein content(μg/10^9^particles)	DNA(μg/10^9^ particles)	RNA(μg/10^9^ particles)
*L. crispatus* BC5-EVs	1.19 ± 0.07	0.22 ± 0.04	0.03 ± 0.01
*L. gasseri* BC12-EVs	1.83 ± 0.27	0.11 ± 0.00	0.08 ± 0.03
MRS-EVs	8.51 ± 0.28	0.28 ± 0.06	0.04 ± 0.01

Data are reported as mean ± standard deviation (SD) (n = 2)

Considering *Lactobacillus*-EVs cargo, protein content was similar in EVs from *L. crispatus* BC5 and *L. gasseri* BC12. Overall, EVs samples contained more DNA than RNA with slight differences in terms of quantity between *Lactobacillus* strains. In particular, EVs from *L. crispatus* BC5 showed numerically higher amount of DNA than *L. gasseri* BC12, while RNA amount was numerically higher in EVs from *L. gasseri* BC12 than *L. crispatus* BC5**.** EVs recovered from sterile culture medium also contain proteins, DNA and RNA in valuable amounts.

### Effects of EVs on *Lactobacillus* adhesion to HeLa cells

**Fig. 1:**
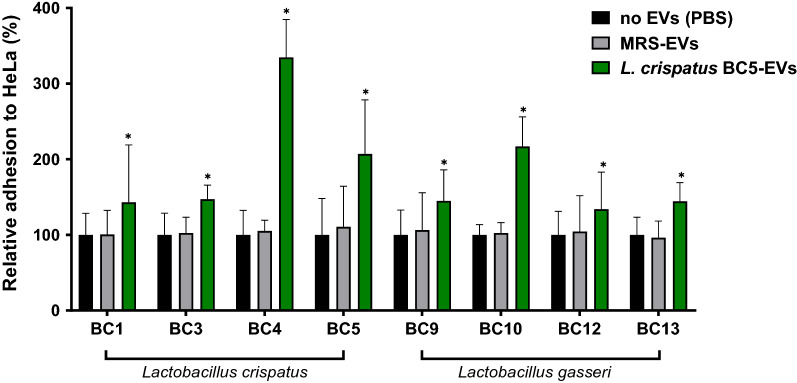
Adhesion assays of *Lactobacillus* strains to HeLa cells in presence of *L. crispatus* BC5-EVs. The adhesion rates are shown as a percentage relative to bacterial adhesion in the absence of EVs (PBS, 100%). Data are reported as mean ± SD (n = 2). *p value < 0.05

**Fig. 2:**
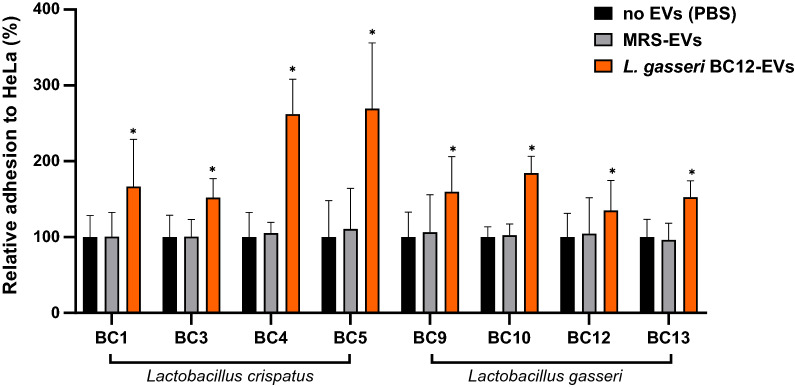
Adhesion assays of *Lactobacillus* strains to HeLa cells in presence of *L. gasseri* BC12-EVs. The adhesion rates are shown as a percentage relative to bacterial adhesion in the absence of EVs (PBS, 100%). Data are reported as mean ± SD (n = 2). *p value < 0.05

To study the effect of EVs in supporting resident lactobacilli colonization, different vaginal *Lactobacillus* strains were allowed to adhere to HeLa cells in the presence of *L. crispatus* BC5-EVs and *L. gasseri* BC12-EVs and results were shown in Figs. 1 and 2, respectively. Bacterial adhesion in the absence of EVs (Phosphate-buffered saline, PBS) was used to normalize data.

First, we excluded a possible cytotoxic effect of EVs on the cell line by the evaluation of HeLa cells morphology and integrity at the optical microscope (data not shown).

EVs effect was evaluated on the adhesion of the producer itself and of other strains belonging to the same species, namely *L. crispatus* BC1, BC3, BC4 and *L. gasseri* BC9, BC10, BC13. As shown in Figs. 1 and 2, the adhesion of *Lactobacillus* strains to epithelial cells significantly increased in the presence of *L. crispatus* BC5-EVs and *L. gasseri* BC12-EVs. Contrariwise, no effect on *Lactobacillus* adhesion was registered in the presence of EVs derived from MRS medium, suggesting that the effect exerted on *Lactobacillus* was specifically associated to EVs origin.

Overall, according to Fig. 1, *L. crispatus* BC5-EVs exerted a good stimulatory activity towards lactobacilli, with an average adhesion ranging from 134% (*L. gasseri* BC12) to 335% (*L. crispatus* BC4). Interestingly, *L. crispatus* BC4 was the most stimulated strain by *L. crispatus* BC5-EVs compared to all strains (average adhesion 335%), including *L. crispatus* BC5 itself (207%). In addition, the highest stimulation activity of *L. crispatus* BC5-EVs towards *L. gasseri* species was observed on strain *L. gasseri* BC10, reaching 217% of average adhesion (ANOVA, *p* < 0.05).

Regarding *L. gasseri* BC12-EVs, as reported in Fig. 2, a similar stimulatory activity was registered, reaching an average adhesion between 135% (*L. gasseri* BC12) and 269% (*L. crispatus* BC5). Particularly, *L. gasseri* BC12-EVs increased more the adhesion of *L. crispatus* BC4 (262%) and *L. crispatus* BC5 (269%) compared to the other strains, including the producer’s itself (135%) (ANOVA, *p* < 0.05).

Considering differences in activity between *L. crispatus* BC5-EVs and *L. gasseri* BC12-EVs, we observed that *L. crispatus* BC5-EVs were significantly more active than *L. gasseri* BC12-EVs regarding the adhesion of only two strains out of eight: *L. crispatus* BC4 (335% and 262%) and *L. gasseri* BC10 (217% and 184%), while *L. crispatus* BC5 resulted to be more stimulated by *L. gasseri* BC12-EVs rather than its own EVs (269% and 207%) (Student’s t-test, *p* < 0.05).

**Fig. 3:**
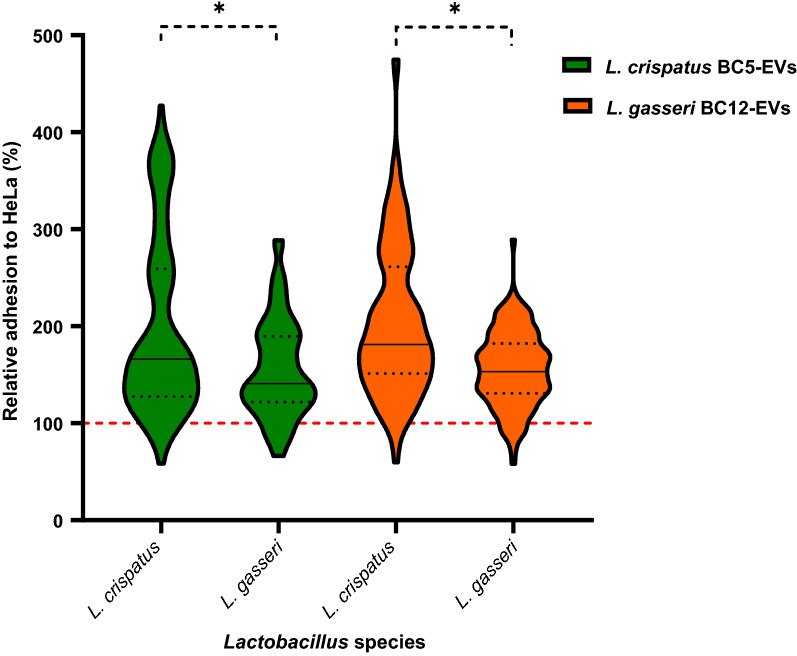
Violin plot of *Lactobacillus* adhesion grouped for species in presence of *L. crispatus* BC5-EVs (green) and *L. gasseri* BC12-EVs (orange). The adhesion rates are shown as a percentage relative to bacterial adhesion in the absence of EVs (PBS, 100%, red line). Solid and dotted black lines represent median values and quartiles, respectively. *p < 0.05

In addition, to investigate the role of EVs in species communication, *Lactobacillus* strains were grouped per species and the adhesion in the presence of *L. crispatus* BC5-EVs and *L. gasseri* BC12-EVs was reported in a violin plot (Fig. 3). Interestingly, EVs derived from *L. crispatus* BC5 and *L. gasseri* BC12 strains mainly affected the adhesion of *L. crispatus* strains than *L. gasseri* ones, pointing out that *L. crispatus* species was more sensitive to the stimulation effect of *Lactobacillus*-EVs. At the same time, no significant differences were found between *L. crispatus* BC5-EVs and *L. gasseri* BC12-EVs regarding the adhesion of *L. crispatus* or *L. gasseri* species*, *showing that EVs effect was not related to the producer species.

### Effects of EVs on pathogens adhesion to HeLa cells

*Lactobacillus crispatus* BC5-EVs and *L. gasseri* BC12-EVs effects were also sought on the adhesion of four common vaginal pathogens, namely *E. coli, S. aureus, S. agalactiae,* and *E. faecalis*.

**Fig. 4:**
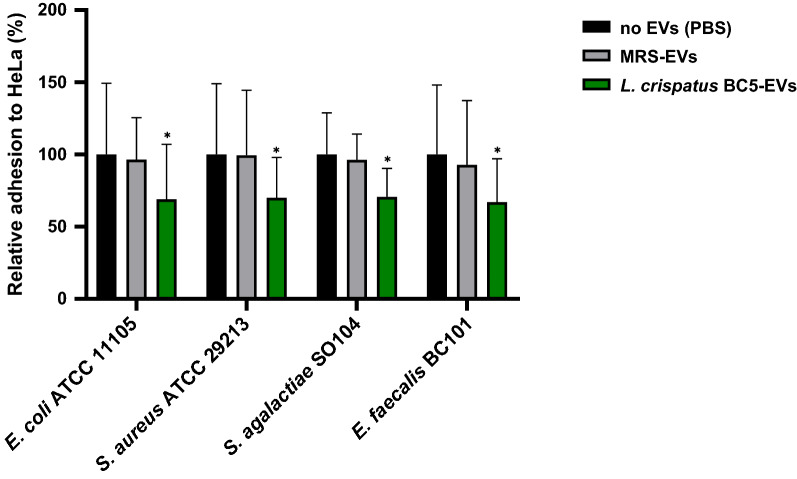
Adhesion assays of *Escherichia coli* ATCC 11105*, Staphylococcus aureus* ATCC 29213*, Streptococcus agalactiae* SO104*,* and *Enterococcus faecalis* BC101 to HeLa cells in presence of *L. crispatus* BC5-EVs. The adhesion rates are shown as a percentage relative to adhesion in the absence of EVs (PBS, 100%). Data are reported as mean ± SD (n = 2). *p value < 0.05

**Fig. 5:**
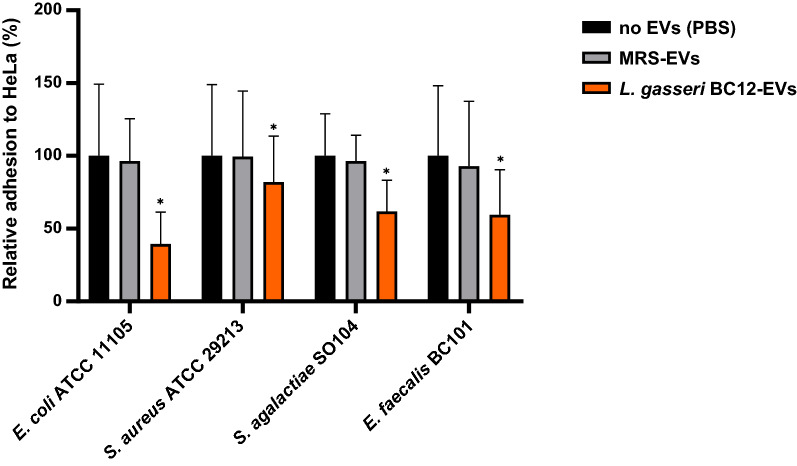
Adhesion assays of *Escherichia coli* ATCC 11105*, Staphylococcus aureus* ATCC 29213*, Streptococcus agalactiae* SO104*,* and *Enterococcus faecalis* BC101 to HeLa cells in presence of *L. gasseri* BC12-EVs. The adhesion rates are shown as a percentage relative to adhesion in the absence of EVs (PBS, 100%). Data are reported as mean ± SD (n = 2). *p value < 0.05

As reported in Figs. 4 and 5, EVs isolated from *L. crispatus* BC5 and *L. gasseri* BC12 significantly reduced the adhesion of all pathogens tested. On the other hand, no activity was observed for EVs isolated from MRS medium, indicating that the inhibitory effect of vesicles was related to EVs origin.

Particularly, EVs from *L. crispatus* BC5 reduced the adhesion of all tested pathogens with similar efficiency, resulting in an average adhesion of 67–71% (Fig. 4). A good anti-adhesive effect was also identified for *L. gasseri* BC12-EVs, with slight differences in pathogens’ adhesion depending on the strain tested (39–82%) (Fig. 5).

Considering differences in activity between *L. crispatus* BC5-EVs and *L. gasseri* BC12-EVs, we observed that *L. gasseri* BC12-EVs were significantly more active than *L. crispatus* BC5-EVs in reducing the adhesion of only one strain out of four: *E. coli* (39% and 69% of average adhesion, respectively) (Student’s t-test, *p* < 0.05). These results indicated that the inhibitory effect of EVs was not related to *Lactobacillus* producer strains, rather, it was related to pathogens’ strain sensitivity.

## Discussion

The vaginal microbiota of healthy reproductive-age women is characterized by the abundance of the *Lactobacillus* genus, being *L. crispatus, L. gasseri, L. jensenii,* and *L. iners* the most common species [1]. *Lactobacillus* spp. play an important role in maintaining the woman’s state of health by regulating the microbiota homeostasis and reducing pathogens adhesion, proliferation, and consequent infections [21].

Recently, it was discovered that vaginal *Lactobacillus* strains release nanosized membrane particles, named extracellular vesicles (EVs), implicated in cell-to-cell and microbiota-host communications [8]. According to literature, EVs play a crucial role in a variety of physiological and pathological processes due to their capacity to carry bioactive macromolecules (i.e., proteins, DNA, and RNA) that can alter the biological properties of bacteria and/or host cells [22].

Compared to other human niches, only a few studies have been carried out on EVs released in the vaginal environment by beneficial lactobacilli, and our understanding of their biogenesis, composition, and functionality are still poor [22, 23].

Here, we evaluated for the first time the contribution of EVs released by vaginal lactobacilli in maintaining the microbiota balance by modulating the microorganism’s colonization. In particular, we investigated the ability of *Lactobacillus*-EVs to promote the adhesion of lactobacilli to HeLa cells and their anti-adhesive effect towards pathogens’ attachment.

EVs were recovered from two vaginal strains, belonging to *L. crispatus* and *L. gasseri* species, frequently predominant in a healthy vaginal microbiota [1]. We observed that *L. crispatus* BC5 and *L. gasseri* BC12 released nanosized vesicles, whose size and concentration were coherent with those previously reported by Palomino et al. [8]. Moreover, the dimensions of EVs from vaginal *Lactobacillus* were comparable to those of EVs released by other lactobacilli, isolated from different human niches (50–200 nm) [12, 16, 17, 19, 24, 25]. Regarding EVs surface charge, *L. crispatus* BC5 and *L. gasseri* BC12-EVs presented negative values of Z-potential (− 20.3 mV and  − 10.4 mV, respectively), with differences between lactobacilli strains. These values are in agreement with those reported for other EVs from Gram-positive bacteria, i.e., *Lactobacillus casei, Bacillus subtilis* and *Bacillus anthracis,* whose Z-potential values are − 8.7 mV, − 18.2 mV and − 65.6 mV, respectively [17, 26]. Since EVs are structures mainly composed by negative charged phospholipids, as exosomes from eukaryotic cells, a negative value of Z-potential is expected [27]. Here, we also recovered EVs from MRS medium, but in concentration 2.5-fold lower than those reported for *Lactobacillus* samples. MRS-EVs average size were similar to those reported by Palomino et al. [8] (150–160 nm) and characterized by a negative Z-potential value. Since it is generally recognized that bacterial EVs effect can be associated to their cargo [22], we decided to characterize *Lactobacillus*-EVs composition in terms of total protein and nucleic acids content. As previously reported by Palomino et al. [8], we confirmed that *L. crispatus* BC5-EVs and *L. gasseri* BC12-EVs contain proteins and, for the first time, also DNA and RNA were recovered. Particularly, the protein content was higher than the content of DNA and RNA, in accordance with previous studies regarding vesicles from other *Lactobacillus* strains [17, 28]. Considering the content of nucleic acids, DNA and RNA were found in both *L. crispatus* BC5-EVs and *L. gasseri* BC12-EVs samples with slight differences in concentration between the two. The DNA concentration was higher in *L. crispatus* BC5-EVs sample, while RNA concentration was higher in *L. gasseri* BC12-EVs compared to *L. crispatus* BC5-EVs one. Also, EVs from MRS medium displayed to contain proteins and nucleic acids. This result is not surprising since MRS is a complex medium containing high amounts of digests, i.e. peptone, yeast extract and beef extract. From our findings, it appears that the biological macromolecules of medium ingredients are also carried by nanometric vesicles. As far as we know, there is no characterization of nucleic acids delivered by *Lactobacillus*-EVs and further studies are required to deeply analyze EVs composition [22].

The effect of EVs was studied on *Lactobacillus* adhesion considering the producer strains themselves and other strains belonging to the same species. We demonstrated that EVs released by *L. crispatus* BC5 and *L. gasseri* BC12 stimulated the adhesion of all *Lactobacillus* in a similar way, with some variability among strains. Notably, no effect on *Lactobacillus* adhesion rates was induced by MRS-EVs, pointing out that the observed activity was related to *Lactobacillus* EVs peculiarities rather than the mere presence of nanometric EVs, that have been also retrieved in MRS medium.

Interestingly, considering *Lactobacillus* species, *L. crispatus* adhesion was more stimulated by both *L. crispatus* BC5-EVs and *L. gasseri* BC12-EVs than *L. gasseri* adhesion, underlying that the stimulation effect of EVs was not related to the producer strain, on the contrary, different *Lactobacillus* species may have different sensitivity to EVs modulation.

The vaginal tract is naturally characterized by the coexistence of different *Lactobacillus* species*,* with the prevalence of one above the others [1]. Due to this co-inhabitance, lactobacilli have adopted a collaborative strategy rather than promoting competitive behavior to survive. Particularly, coculture experiments of *L. crispatus* and *L. gasseri* species demonstrated a cooperative behavior between the species in terms of niche colonization but *L. crispatus* species better colonizes the niche compared to *L. gasseri*, underlying the high adaptability of this species to others favoring its persistence [29].

It has been reported that *Lactobacillus*-EVs metabolites, nucleic acids, and protein content can be associated to a peculiar biological role of EVs [18, 22]. Regarding *L. crispatus* BC5-EVs and *L. gasseri* BC12-EVs protein content, Palomino et al. identified some adhesins (i.e., enolases, elongation factor-TU, 30S ribosomal protein, pyruvate kinase, chaperon proteins) involved in *Lactobacillus* attachment to human cell receptors [8, 30–32]. Moreover, EVs from *L. casei* delivered an exclusive adhesin protein absent in cell extracts, suggesting that EVs may affect *Lactobacillus*-host cell interfaces [17].

Beneficial effect exerted by lactobacilli is, at least in part, related to the ability of reducing pathogens adhesion to host cell surfaces [5, 33]. In this regard, supernatants from *Lactobacillus* were found to reduce pathogens adhesion, suggesting that *Lactobacillus* derivatives can exert an anti-adhesive activity [34].

Here, we hypothesized that EVs could mediate *Lactobacillus* anti-adhesive properties in the vaginal ecosystem. *Lactobacillus*-EVs were evaluated towards the adhesion of four vaginal opportunistic pathogens, i.e., *E coli, S. aureus, E. faecalis,* and *S. agalactiae*. Surprisingly, *L. crispatus* BC5-EVs and *L. gasseri* BC12-EVs were able to reduce the adhesion of all pathogens tested with similar effects.

As reported above, the EVs inhibitory mechanisms can be associated to some *Lactobacillus* adhesins found in *L. crispatus* BC5-EVs and *L. gasseri* BC12-EVs by Palomino et al. [8]. These adhesins are involved not only in *Lactobacillus* adhesion but also in preventing pathogens’ interactions to host receptors by competitive inhibition [31, 35]. In particular, EVs from *L. crispatus* BC5 and *L. gasseri* BC12 delivered enolase-1 that resulted to reduce *Neisseria ghonorreae* attachment to host cells [8, 36, 37]. Moreover, *L. gasseri* BC12-EVs transported two more adhesins (i.e., enolase-2 and elongation factor-TU) that inhibited *E. coli* adhesion to host mucosa [8, 35, 38]. Our data, supported by literature, suggested that *Lactobacillus*-EVs could prevent pathogens adhesion by the saturation of host adhesins receptors.

Besides these suggestions, many more aspects of *Lactobacillus*-EVs activity might be elucidated. In this regard, a possible effect of EVs on cervical cells can also be considered. Until now, it is known that EVs mediate the *Lactobacillus* crosstalk communication with the human host cells and trigger some cell signaling cascades, specially related to the host immune system [12, 13, 18, 19, 23, 24, 28]. Previous studies reported that vaginal lactobacilli were able to modify HeLa cell plasma membrane in terms of lipid composition, fluidity, and protein exposure, making the host less permissive to *Candida albicans* and *Chlamydia trachomatis* attachment and infection [39, 40]. Moreover, as well as EVs from pathogenic bacteria, EVs from beneficial *Lactobacillus* can deliver DNA and RNA to host cells, possibly affecting gene expression [41–43]. Even if the molecular mechanism is not fully understood, since EVs cargo is composed of molecules from different cell compartments, we can’t exclude possible EVs-induced modifications on host pathways [44]. In this respect, a modification in membrane fluidity and/or gene expression in host cells could alter the rotational and lateral motion and/or expression of receptors affecting their availability for bacterial recognition [45].

Whether the effect of *Lactobacillus*-EVs on bacteria adhesion is the result of one or a combination of the mechanisms proposed remains an open and, in some ways, tough question to answer. A deeper characterization of *Lactobacillus*-EVs structure and the purification of EVs components could allow a wider understanding of EVs mode of action. In this perspective, we are planning to further characterize EVs physical and chemical properties by super-resolution and electron microscopy, as well as the nucleic acids quality and integrity by high-resolution sequencing techniques.

As a matter of fact, our discovery provides new insights into the role of *Lactobacillus*-EVs in modulating the vaginal ecosystem and gives further information on their functionality within this ecological niche.

In this perspective, our results reinforce the association between *Lactobacillus* EVs and health benefits [8, 15, 18, 20], opening to the idea of using EVs derived from vaginal strains as potential postbiotics to support the vaginal balance in favor of the host well-being.

## Methods

### Bacterial cultures and growth conditions

*Lactobacillus* strains used in this study were previously isolated from vaginal swabs of healthy premenopausal women, according to the protocol of the Ethics Committee of the University of Bologna (52/2014/U/Tess) [10]. Here, we selected strains belonging to two species highly represented in the vaginal niche: *L. crispatus* (BC1, BC3, BC4, and BC5) and *L. gasseri* (BC9, BC10, BC12, and BC13). *Lactobacillus* strains were cultured anaerobically at 37 °C in de Man, Rogosa, and Sharpe (MRS) (Beckton, Dickinson, and Co., MI, Italy) broth with the supplement of 0.05% L-cysteine (Sigma-Aldrich, MI, Italy). The anaerobic conditions were reached through jars containing GasPak EZ (Beckton, Dickinson, and Co.).

Pathogenic bacteria used for the anti-adhesive study were *Escherichia coli* ATCC 11105*, Staphylococcus aureus* ATCC 29213*, Streptococcus agalactiae* SO104*,* and *Enterococcus faecalis* BC101*. E. coli* and *S. aureus* were cultured aerobically in Nutrient Broth (NB) (Beckton, Dickinson, and Co.) at 37 °C. *S. agalactiae* SO104 was isolated from vaginal swabs in the Microbiology Laboratory of Sant’ Orsola-Malpighi University Hospital of Bologna (Italy) during routine diagnostic procedures. *E. faecalis* BC101 belongs to the Department of Pharmacy and Biotechnology, University of Bologna (Italy) [11]. *S. agalactiae* and *E. faecalis* were cultured in Brain Heart Infusion (BHI) (Beckton, Dickinson, and Co.) broth, in 5% CO_2_ at 37 °C.

For each microorganism, two sequential 24 h-cultures were carried out, then 1 × 10^9^ CFU/mL bacterial suspensions were prepared in sterile saline and used in adhesion assays.

### Isolation of extracellular vesicles (EVs) from *Lactobacillus*

EVs were recovered from *L. crispatus* BC5 and *L. gasseri* BC12 growth cultures. EVs were isolated according to Ñahui Palomino et al. 2019, with some modifications [8, 46]. MRS medium and phosphate-buffered saline pH 7.4 (PBS) used for EVs isolation were previously autoclaved (120 °C for 30 min) and filtered with 0.22 μm polyethersulfonate PES vacuum filters (Membrane Solutions, LLC, Auburn, WA, USA) to remove large particles and possible contaminants. *Lactobacillus* were cultured anaerobically for 24 h in filtered MRS and then subcultured in fresh medium for additional 24 h. 200 mL of bacterial suspensions (1 × 10^9^ CFU/mL) were centrifuged at 3600×*g* for 15 min at 4 °C (Sartorius Centrisart^®^ D-16C, Sartorius, Goettingen, Germany). Supernatants were collected and subsequently filtered with 0.22 μm cellulose acetate filters to eliminate any remaining bacteria. Afterward, the filtered supernatants were centrifugated at 10000×*g* for 30 min to eliminate any cell debris. EVs were precipitated from obtained supernatants by ultracentrifugation at 100,000×*g* for 70 min at 4 °C (Beckman Optima L-90 K, Rotor: SW 28 Ti Swinging-Bucket, capacity 8 × 38.5 mL, Beckman Coulter, Inc., Brea, USA) and washed with PBS using the same centrifugation setting. EVs pellet was resuspended in PBS (final volume of 1.5 mL) and stored at − 80 °C until use. The same protocol was applied to isolate particles from filtered MRS medium.

### Physical and chemical characterization of EVs

EVs samples were characterized for their physical properties in terms of yield and size by Nanoparticle Tracking Analysis (NTA) technology (NanoSight 49 NS300, Malvern Panalytical, Grovewood Road, Malvern, UK). Samples were diluted at 1:100 in PBS and videos were recorded at 30 frames per second using a 20× objective. Measurements of EVs Zeta-potential were performed with the instrument Malvern Zetasizer 3000 HS (Malvern Panalytical Ltd., Malvern, UK) set at 25 °C by diluting EVs samples 1:2 in a MilliQ water. EVs protein content was measured by Bradford assay (BioRad Laboratories, Inc., CA, USA) after EVs lysis. EVs were lysed with RIPA buffer according to Prabal Subedi et al., with some modifications [47]. RIPA buffer 5× was prepared as follows: 50 mM Tris–HCl (pH 8.0), 5 mM EDTA, 2.5 mM EGTA, 5% Triton X-100, 0.5% sodium deoxycholate, 0.5% SDS, 140 mM NaCl, aliquoted and stored at − 20 °C until use. Briefly, 10 μL of EVs suspension were incubated with 2.5 μL of RIPA 5× at 4 °C for 30 min, afterwards, samples were placed in an ice-cold sonication bath for 30 s. This step was followed by a gentle agitation on ice for 15 min. Protein concentration was considered to normalize EVs treatments in adhesion assays.

DNA and RNA were isolated from EVs samples through TRIzol™ Reagent (Invitrogen, Thermo Fisher Scientific Inc, Massachusetts, USA) according to the manufacturer’s instructions. Before the isolation procedure, approximately 1.5 mL of EVs were pelleted as previously described and concentrated in a volume of 500 μL. EVs were treated with 1.5 mL of TRIzol™ Reagent and the manufacturer’s protocol was followed. Briefly, after the addition of TRIzol™ Reagent, 300 μL of chloroform were added and samples centrifuged for 15 min at 12,000×*g* at 4 °C. The mixture separated into a lower red phenol–chloroform, a gel interphase and a colorless aqueous upper phase. The DNA was extracted from the interphase and the lower phase while the RNA was recovered from the aqueous phase. The DNA was precipitated in ethanol 100% (v/v), washed once in 0.1 M sodium citrate and resuspended in ethanol 75% (v/v). Afterwards, the DNA was pelleted and resuspended in 200 μL of 8 mM NaOH, 1 mM EDTA and then buffered to pH 7.0 with 0.1 M HEPES. Starting from the aqueous phase, RNA was precipitated in isopropanol 99% (v/v) and resuspended in ethanol 75% (v/v). Afterwards, the RNA was pelleted, resuspended in 20 μL of 0.1 mM EDTA and incubated for 15 min in a 55 °C water bath, to allow RNA solubilization. Samples of DNA were stored at − 20 °C while RNA was stored at − 80 °C. DNA and RNA quantification was assessed by NanoDrop ND-1000 spectrophotometer (NanoDrop Technologies, Wilmington, DE, USA). The contents of protein, DNA and RNA were normalized on 1 × 10^9^ particles.

### Cell cultures

HeLa cell line was routinely grown in 25 cm^2^ tissue culture flasks, at 37 °C in 5% CO_2_ in Dulbecco’s Modified Eagle medium (DMEM, Lonza Group Ltd, Basel, Switzerland) supplemented with 10% Fetal Serum Bovine (FBS) and 1% l-glutamine. For adhesion experiments, cells were seeded at a density of 2 × 10^4^ cells/cm^2^ on sterile round coverslips in 24-wells cell culture plates (Sarstedt AG & Co., Nümbrecht, Germany) and allowed to grow to 80% confluence (approx. 3 days). Before adhesion assay, exhausted medium was replaced with fresh complete medium (0.2 mL per well).

### Adhesion assays

A certain volume of *Lactobacillus* EVs corresponding to 20 μg of proteins was used to pretreat 1 × 10^5^ HeLa cells for 1 h, in 5% CO_2_ at 37 °C. Afterwards, bacterial suspensions (1 × 10^9^ CFU/mL in sterile saline) were added to HeLa monolayer, applying a ratio of 100:1 (bacteria: HeLa cells), and plates were incubated in 5% CO_2_ at 37 °C for an additional hour. Hela cells pretreated with PBS were used to evaluate basal bacterial adhesion (100%). For each sample, at least two independent experiments were carried out. Bacteria adherent to HeLa cell monolayers were stained by May-Grunwald/Giemsa protocol as previously reported [10]. Adherent bacteria were counted at optical microscope Nikon Eclipse 21 (Objective 100×, Nikon, Tokyo, Japan) considering at least 30 microscopic fields per sample and adhesion was expressed as the percentage of adherent bacteria compared to the control.

### Statistical analysis

Statistical analysis was performed using Student’s t-test for two means comparison and ordinary one-way ANOVA for multiple comparisons (GraphPad Prism version 8.0.1, GraphPad Prism Software Inc, San Diego, CA, USA). Results were expressed as mean ± standard deviation (SD) and differences were deemed significant for *p* < 0.05.

## Data Availability

All data generated or analyzed during this study are included in this published article [and its additional files].
